# Electrical impedance tomography in acute respiratory distress syndrome

**DOI:** 10.1186/s13054-018-2195-6

**Published:** 2018-10-25

**Authors:** M Consuelo Bachmann, Caio Morais, Guillermo Bugedo, Alejandro Bruhn, Arturo Morales, João B Borges, Eduardo Costa, Jaime Retamal

**Affiliations:** 1 Departamento de Medicina Intensiva, Facultad de Medicina, Pontificia Universidad Católica de Chile, Santiago, Chile; 2 Acute Respiratory and Critical Illness Center (ARCI), Santiago, Chile; 3 Departamento Enfermedades Respiratorias, Facultad de Medicina, Pontificia Universidad Católica de Chile, Santiago, Chile; 4 Divisao de Pneumologia, Instituto do Coracao (Incor), Hospital das Clínicas, Faculdade de Medicina, Universidade de São Paulo, São Paulo, Brazil; 5 Hedenstierna Laboratory, Department of Surgical Sciences, Section of Anaesthesiology and Critical Care, Uppsala University, Uppsala, Sweden

**Keywords:** Electrical impedance tomography, Acute respiratory distress syndrome, Mechanical ventilation, Ventilation distribution, Lung imaging

## Abstract

Acute respiratory distress syndrome (ARDS) is a clinical entity that acutely affects the lung parenchyma, and is characterized by diffuse alveolar damage and increased pulmonary vascular permeability. Currently, computed tomography (CT) is commonly used for classifying and prognosticating ARDS. However, performing this examination in critically ill patients is complex, due to the need to transfer these patients to the CT room. Fortunately, new technologies have been developed that allow the monitoring of patients at the bedside. Electrical impedance tomography (EIT) is a monitoring tool that allows one to evaluate at the bedside the distribution of pulmonary ventilation continuously, in real time, and which has proven to be useful in optimizing mechanical ventilation parameters in critically ill patients. Several clinical applications of EIT have been developed during the last years and the technique has been generating increasing interest among researchers. However, among clinicians, there is still a lack of knowledge regarding the technical principles of EIT and potential applications in ARDS patients. The aim of this review is to present the characteristics, technical concepts, and clinical applications of EIT, which may allow better monitoring of lung function during ARDS.

## Background

### Acute respiratory distress syndrome

Acute respiratory distress syndrome (ARDS) is a clinical entity that acutely affects the lung parenchyma, and may be triggered by several predisposing conditions. ARDS is characterized by diffuse alveolar damage, increased pulmonary vascular permeability, increased lung weight, and loss of pulmonary aeration. Clinically, the hallmark of this syndrome is acute hypoxemia with bilateral pulmonary infiltrates on chest radiography that are not fully explained by cardiac abnormalities or hypervolemia [1].

Currently, 10% of patients in intensive care units (ICUs) and 23% of those receiving mechanical ventilation have ARDS [2]. In addition to the high mortality (around 40%) [1, 2], ARDS is accompanied by long-term morbidity such as muscle weakness, cognitive disability, depression, and post-traumatic stress disorder [3].

One of the fundamental features of ARDS is the increase in epithelial and endothelial permeability secondary to the generation of cellular gaps [4]. Inflammatory edema induces airspace instability and regional collapse, which renders the lungs heterogeneously aerated, with a noticeable gradient of collapse toward the dependent areas (superimposed gradient) [5]. The inhomogeneity of the parenchyma in patients with ARDS, when quantified with computerized tomography (CT), correlates with the severity of the syndrome and its associated mortality [6]. The amount of normally aerated tissue in the lungs of patients with diffuse-pattern ARDS varies from 200 to 500 g, comparable to the lung size of a 5-year-old child, which is why the term “baby lung” has been coined [7].

In ARDS, there is a characteristic vertical gradient of lung collapse and/or flooding of dorsal airspaces. As pulmonary circulation is preferably distributed to these same dorsal regions, large areas with a decreased V/Q ratio or true shunt are created. Under these conditions, the applied tidal volume will be directed to the ventral regions, imposing on them large strains and consequently ventilator-induced lung injury (VILI) [8–11], even when protective mechanical ventilation protocols are used [12, 13].

CT is a useful tool in the management and study of patients with ARDS. It has allowed the evaluation of phenomena associated with the development of VILI, such as cyclic opening and closing of airspaces, alveolar overdistension [14], and global and regional strain [15, 16]. In addition, it has provided relevant information for clinical decision-making, such as quantification of the potential for recruitment [17]. However, the use of radiation and the need to transfer the patient to the CT room limits routine use. In this scenario, during the last years, electrical impedance tomography (EIT) has emerged as an important device to monitor and adjust the management of mechanically ventilated patients.

### Concept of electrical impedance tomography

EIT is a noninvasive, radiation-free clinical imaging tool to monitor, in real time and at the bedside, the distribution of ventilation. EIT image reconstruction is based on the estimation of the resistivity changes that occur across the lungs with breathing [18]. The increase in resistivity that occurs with lung inflation is due to the thinning and elongation of alveolar septa, both of which impair the passage of electrical current. Resistivity has been reported to increase more than twofold with deep breaths [19], and correlates closely with the amount of air that enters the lungs. EIT can also track the distribution of pulmonary blood flow, following an intravenous bolus of hypertonic saline.

**Fig. 1:**
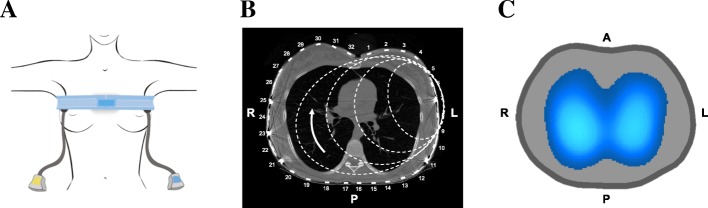
**a** Placement of electrode belt on chest. It is recommended to apply electrode belt between fifth and sixth intercostal space. **b** Computed tomographic axial slice of thorax with 32-electrode belt, and schematic representation of electrical current pathways through thorax. One pair of electrodes injects electrical current while remaining electrodes read voltages produced as a result of the distribution of current density inside thorax. Injection pair is alternated sequentially, and after a full cycle one image will be generated. **c** Functional image reconstructed by electrical impedance tomography (EIT) using a color scale: the lighter the blue, the greater the regional ventilation. Of note, this color scale is not universal. Image generated by EIT Enlight (TIMPEL SA, São Paulo). A anterior, L left, P posterior, R right

The principles of EIT imaging have been described in detail elsewhere [20]. Briefly, small alternating electrical currents are delivered through 8–32 (depending on model and brand) equally spaced electrodes applied circumferentially around the thorax (Fig. 1a). Commonly, one pair of electrodes is used at a time, while the remaining electrodes read the resulting voltages (Fig. 1b). The injection pair is alternated sequentially, and at the end of one full cycle all voltage measurements are used to produce one image, according to specific reconstruction algorithms. Each image frame is generated in comparison to a reference period collected usually at baseline. After reconstruction with a refined finite element mesh, the images are projected into an array of 32 × 32 pixels, where each pixel will describe the resistivity variation over a time interval in relation to a reference moment (Fig. 1c) [21]. Pixels represent changes in relation to this reference, and image frames are usually called relative images. The spatial orientation of the EIT image is similar to that used by CT, with the right side of the chest located to the left of the image and the anterior region at the top of the image (Fig. 1b, c).

**Fig. 2:**
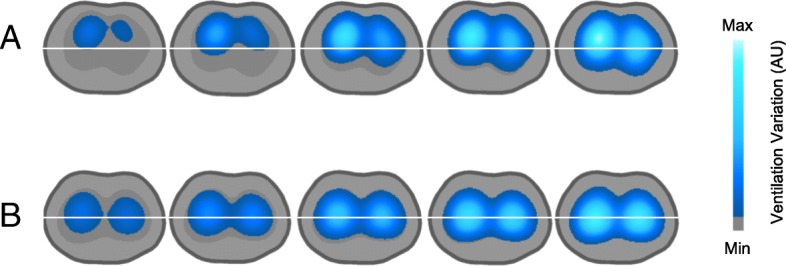
**a** Heterogeneous inflation. Ventral regions inflate first and dorsal regions start inflating halfway to end of inspiration. **b** Homogeneous inflation. Both ventral and dorsal regions start inflating simultaneously. AU arbitrary units

What leverages EIT in relation to other imaging methods is its high temporal resolution. Modern EIT devices generate up to 50 images per second, which allows the dynamic study of ventilation distribution, regional lung perfusion and lung pulsatility. For example, it is possible to show that some areas start to inflate after the others, reflecting either tidal recruitment (Fig. 2) or pendelluft. The downside of the technique is its low spatial resolution, comparable roughly to scintigraphy.

Several studies have shown benefits of using EIT to set ventilatory parameters, improving gas exchange and respiratory mechanics in animal models [22–25]. In the clinical scenario, there is growing evidence that EIT may be a useful tool to optimize individual ventilatory parameters in critically ill patients and potentially reduce the risk of VILI [26, 27]. In the following sections we will describe first the basic tools of EIT, and then the clinical tools with potential application for ARDS.

## Electrical impedance tomography basic tools

### EIT plethysmogram

The EIT plethysmogram is a waveform derived from the sum of all pixels within a given region of interest (ROI) of a relative image (frame) plotted against time. It represents the amount of air that moves in and out of the ROI.

**Fig. 3:**
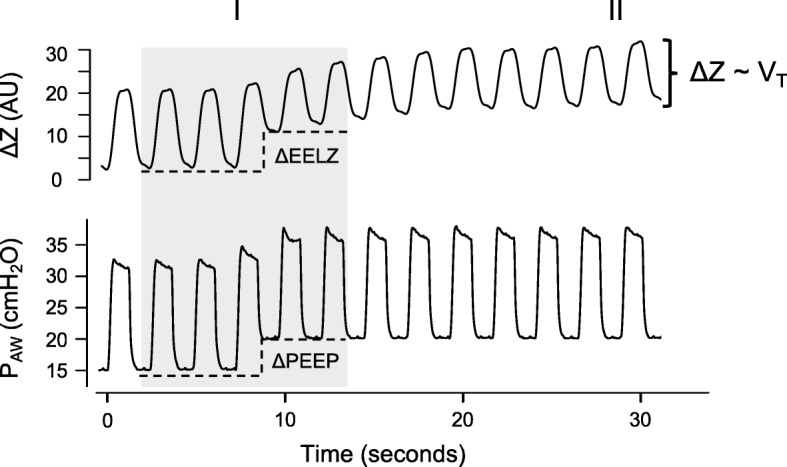
Global (whole image) plethysmogram and airway pressure (*P*_AW_) waveforms. (I) Increment in positive end-expiratory pressure (PEEP) increased end-expiratory lung volume (ΔEELZ). (II) Ventilatory cyclical variation (Δ*Z*) tracks changes in tidal volume (V_T_). AU arbitrary units

The tidal oscillation in the global plethysmogram caused by each respiratory cycle, called Delta *Z* (Δ*Z*), closely correlates with the change in lung volume estimated by CT (*R*^2^ = 0.92) [28]. A strong correlation was also found between the end-expiratory lung volume (EELV), estimated by the multibreath nitrogen-washout maneuver, and the end-expiratory lung impedance (EELZ) (*R*^2^ = 0.95) [29]. Therefore, in addition to monitoring pulmonary ventilation (Δ*Z*), EIT identifies changes in pulmonary aeration (through ΔEELZ) caused, for example, by position changes or positive end-expiratory pressure (PEEP) adjustments (Fig. 3).

### Ventilation map

**Fig. 4:**
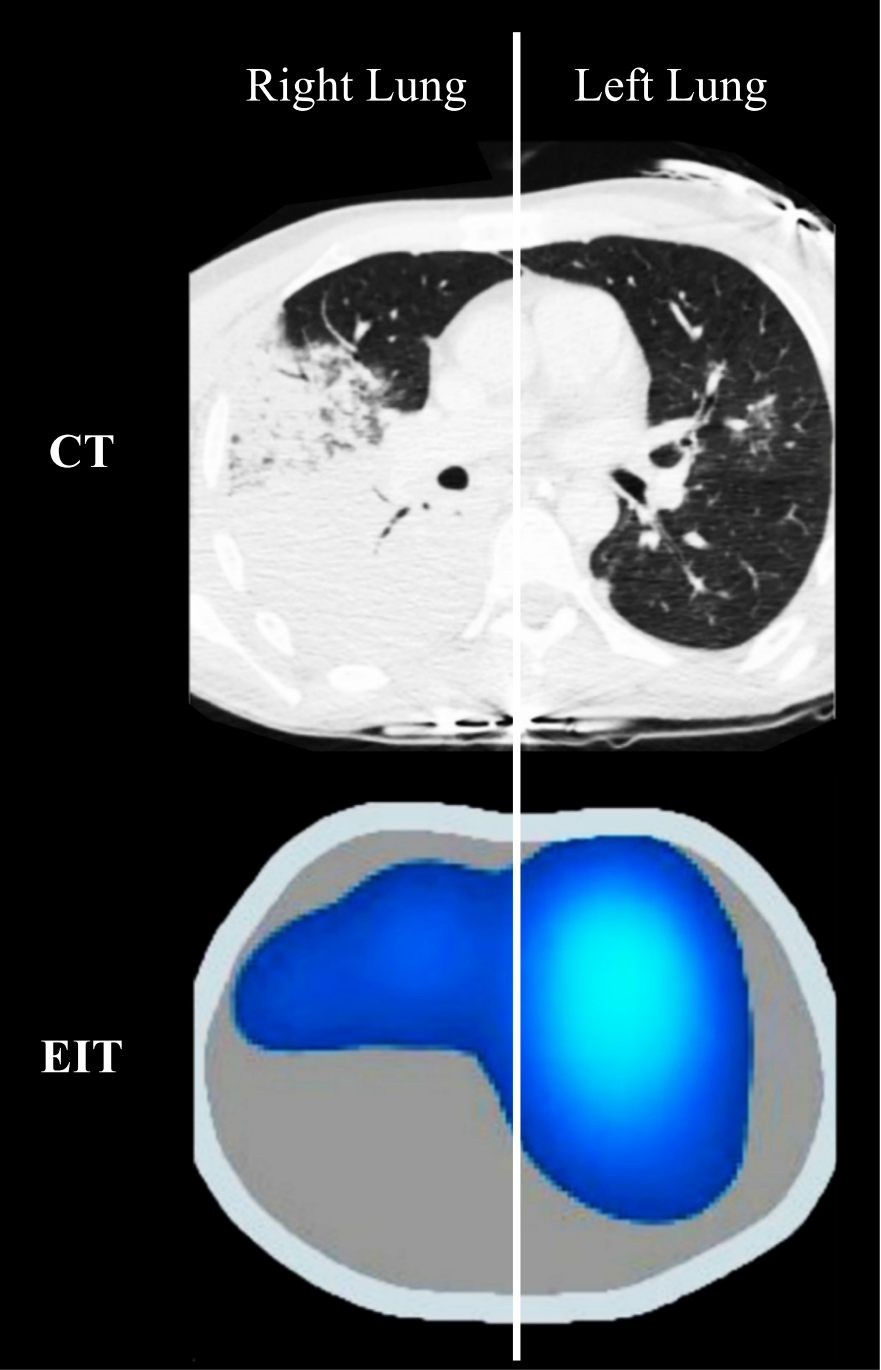
Computed tomography (CT) of a patient with pneumonia and corresponding functional image obtained from electrical impedance tomography (EIT). Note absence of ventilation on lower right lung in EIT image and corresponding massive consolidation on right lung assessed by CT

The ventilation map or functional image is a representation of the tidal changes in impedance pixel by pixel (i.e., it is a color map of the pixelwise Δ*Z*). By positioning horizontal and/or vertical cursors in this functional image, it is possible to quantify the distribution of ventilation in the right-to-left direction, the ventral-to-dorsal direction, or to quadrants. This method has been validated with electron beam CT [30], single photon emission CT (SPECT) [31], and CT images [32], and is commonly used to identify heterogeneities in the distribution of ventilation caused by pathologies and/or ventilatory settings (Fig. 4).

**Fig. 5:**
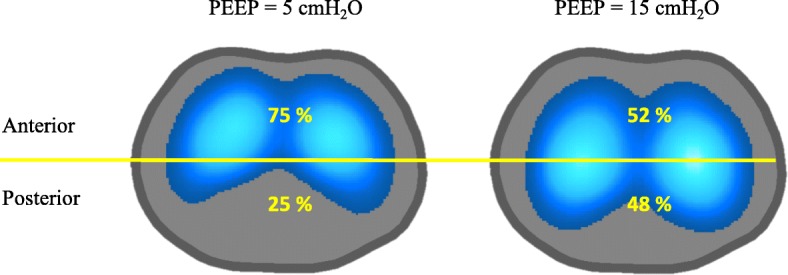
Ventilation map divided into two regions of interest in a model of acute respiratory distress syndrome, ventilated with positive end-expiratory pressure (PEEP) of 5 cmH_2_O (left) and 15 cmH_2_O (right)

Figure 5 exemplifies a functional image divided into two ROIs (ventral and dorsal) in a porcine model of ARDS. Note the heterogeneous ventilation distribution (expressed as a percentage) in the ventral and dorsal regions at PEEP of 5 cmH_2_O. Increasing PEEP to 15 cmH_2_O resulted in a more homogeneous distribution between the regions.

## Clinical tools

### Estimation of lung collapse and overdistension

The ventilation heterogeneity in the ARDS lung is generally associated with the existence of injurious mechanisms, such as the collapse and cyclic opening of small airways and alveoli, and pulmonary overdistension. Costa et al. [33] developed a method to estimate pulmonary collapse and overdistension using regional information (pixel compliance) during a decremental PEEP maneuver. In each PEEP step, the compliance can be calculated from the amount of air entering the lung (Δ*Z*) and from the elastic pressure of the respiratory system; that is, the difference between the plateau pressure (*P*_plateau_) and PEEP. Thus, the compliance of each EIT pixel can be estimated as:

$$ {\mathrm{Compliance}}_{\mathrm{pixel}}=\Delta  Z/\left({P}_{\mathrm{plateau}}-\mathrm{PEEP}\right). $$

**Fig. 6:**
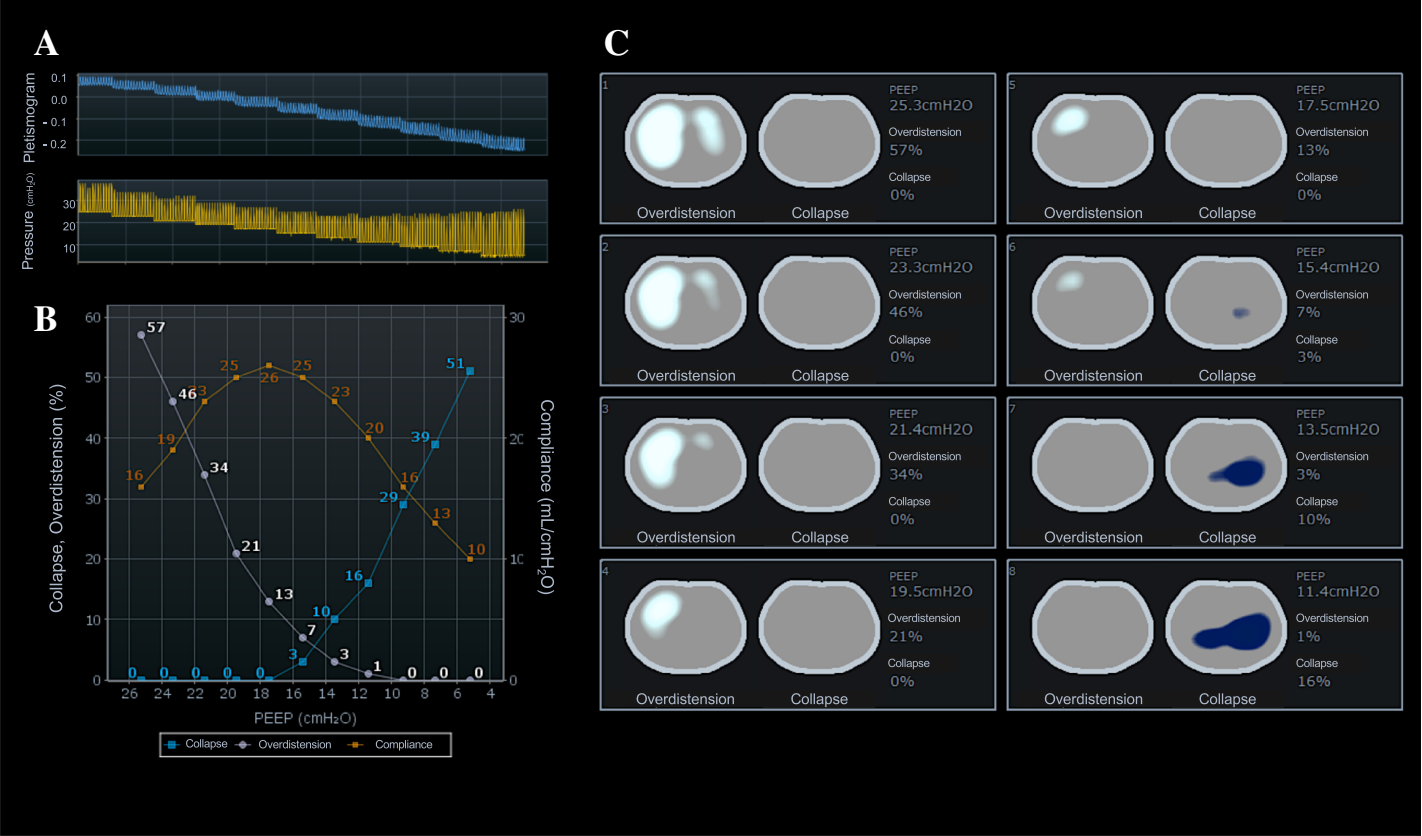
Estimation of recruitable lung collapse and overdistension during decremental positive end-expiratory pressure (PEEP) maneuver. **a** Reduction of end-expiratory lung impedance (blue waves) in each PEEP step (yellow waves). **b** Respiratory system compliance, collapse, and overdistension at each stage of decremental PEEP maneuver. Note that PEEP of better global compliance (17 cmH_2_O) does not coincide with PEEP that minimizes collapse and overdistension estimated according to electrical impedance tomography (15 cmH_2_O). **c** Maps of overdistension and collapse in each PEEP step. Observe progressive increase of lung collapse with reduction of PEEP, predominantly in dependent region. Images generated by Enlight (Timpel SA, São Paulo, Brazil)

This method assumes that loss in pixel compliance at PEEP levels above the PEEP of best pixel compliance indicates overdistension. Similarly, the method assumes that loss in compliance at PEEP levels below the PEEP of best pixel compliance indicates collapse (Fig. 6). The method estimates the amount of recruitable collapse; that is, the amount of recruited lung that is lost following a decremental PEEP trial. When performed right after a recruitment maneuver, this EIT estimation of lung collapse approximates that quantified with CT.

Recently, Beda et al. [34] showed that EIT-derived pressure–volume (PV) curves could identify regions of presumed tidal recruitment and overdistension. Changes in PV shape-derived tidal recruitment were correlated with changes in poorly aerated regions, and changes in PV shape-derived overdistension were highly correlated with changes in hyperaerated regions for higher PEEPs (*r* = 0.73).

Meier et al. [35] used EIT to monitor the regional tidal volume during a PEEP titration maneuver in an experimental model of surfactant depletion. Based on changes in regional ventilation secondary to changes in the PEEP level, the researchers temporarily identified the onset of collapse and regional lung recruitment even before global changes in pulmonary mechanics occurred. These findings were compared with CT images and a good correlation was found between regional volumes of end-expiratory gas and tidal volume estimated by both tools. The authors concluded that EIT is adequate to monitor the dynamic effects of PEEP variations on regional ventilation.

Another interesting application of EIT is the possibility of detecting airway closure, a phenomenon recently described in ARDS patients by Chen et al. [36] when carefully analyzing low-flow pressure–volume curves. They noted that airway closure could be easily missed by clinicians at the bedside. Sun et al. [37] recently presented the case report of a patient with moderate ARDS, in which they evaluated global and regional PV curves, EIT ventilation maps, and plethysmograph waveforms during low-flow inflation, finding that EIT-derived regional PV curves might be a useful method to confirm the presence of the airway occlusion phenomenon.

### Pneumothorax detection

**Fig. 7:**
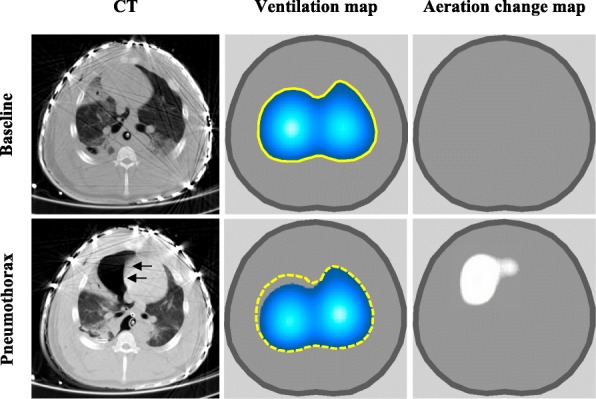
Computed tomography (CT), ventilation map, and aeration change map obtained at baseline and after induction of pneumothorax in a pig. Arrows point to accumulation of air in pleural space

The incidence of pneumothorax in patients with ARDS is 8–10% [38]. EIT has been used as a bedside tool to detect the presence of pneumothorax in real time. In 2006, Hahn et al. [39] studied through an experimental model the changes in the EIT images by inducing variable degrees of pneumothorax. They found an increase in the impedance in the aeration map (static change) associated with a decrease in regional ventilation (dynamic change). These findings were compared with CT images, demonstrating the ability of EIT to detect pneumothorax in real time. Costa et al. [40] confirmed in an experimental model that EIT is able to detect the presence of pneumothorax in real time (three respiratory cycles of delay) with 100% sensitivity (Fig. 7).

Recently, Morais et al. [41] described a case of pneumothorax appearance with EIT as a complication of a lung recruitment maneuver performed late in the course of ARDS. In this case, the EIT changes induced by the pneumothorax (sudden increase in brightness in the EIT image, and the increase in aeration out of proportion to the increase in PEEP) led to early interruption of the recruitment maneuver before the onset of clinical deterioration. This is an example of how EIT monitoring can help manage patients with severe ARDS submitted to procedures involving risk for barotrauma, such as lung recruitment maneuvers.

### Monitor the effects of endotracheal aspiration on pulmonary volumes

**Fig. 8:**
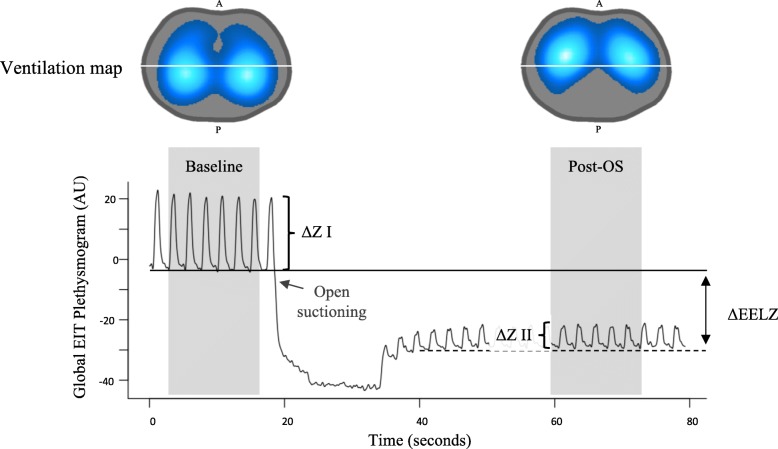
Global electrical impedance tomography (EIT) plethysmogram and ventilation map during open suction (OS) in model of severe ARDS. Solid and dotted horizontal lines represent end-expiratory lung impedance (EELZ) at baseline and post OS, respectively. Note that EELZ does not return to baseline values (arrows indicating distance between solid and dotted lines), describing reduction of aerated lung. Also note reduction of pulmonary ventilation after OS (Δ*Z* I – Δ*Z* II). Ventilation maps I and II (left and right images at top) show decrease of ventilation on posterior region after OS. A anterior (ventral), AU arbitrary units, P posterior (dorsal). Courtesy of Nadja Carvalho

Another utility of EIT is to identify the effects of endotracheal suction on pulmonary volumes. Lindgren et al. [42] evaluated lung volume changes by EIT during endotracheal suction in an experimental model of surfactant depletion, and showed a greater alveolar collapse, especially in the dorsal regions of the lung. Approximately 50% of the functional residual capacity (FRC) was lost after disconnection of the tube and 20% more at the time of suction [42]. In postoperative cardiac patients, EELZ remained at values below the presuction intervention, even 30 min after restoring mechanical ventilation [43]. Figure 8 shows the effect of open suctioning on lung volumes in a model of severe ARDS. Note the marked reduction of EELZ and ∆*Z* after the suction procedure. The maps of ventilation indicate an inversion on the ventilation distribution between the ventral and dorsal regions after the open suctioning.

### Ventilatory dyssynchrony

Patient–ventilator dyssynchronies are common during mechanical ventilation and are usually related to adverse events such as weaning prolongation and increased mortality [44]. Despite this, the vast majority of dyssynchronies (more than 60%) remain undetected by experts on inspection of ventilator waveforms [45]. In this scenario, the information contained in the EIT plethysmogram may assist the intensivist in the early identification of potentially harmful dyssynchronies, such as breath stacking and pendelluft.

**Fig. 9:**
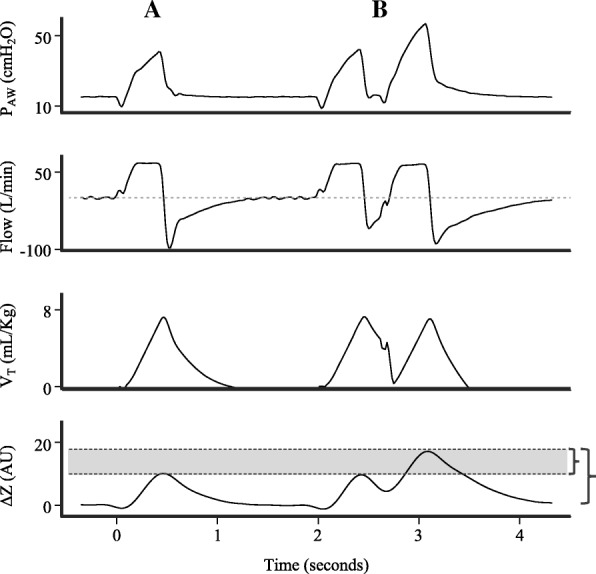
Airway pressure (P_AW_), flow, tidal volume (V_T_), and EIT waveforms during synchronous cycle (A) and during breath stacking dyssynchrony (B). During breath stacking, plethysmogram shows inspired volume near twice that of a regular cycle. This excessive deformation of lung not detected by currently available waveforms on mechanical ventilators. AU arbitrary units, ∆*Z* variation of impedance

Breath stacking is usually secondary to reverse triggering or double-triggering, when a second respiratory cycle is imposed by the ventilator on top of an incomplete exhalation [46]. Figure 9 shows airway pressure, flow, and volume (found in the mechanical ventilator) and EIT waveforms during a synchronous cycle (A) and during breath stacking dyssynchrony (B). During breath stacking, the volume waveform shows an inspired volume of approximately 8 ml/kg of predicted weight; however, the inspired volume detected by EIT is nearly twice that of a regular cycle, which indicates injurious deformation of the lung.

**Fig. 10:**
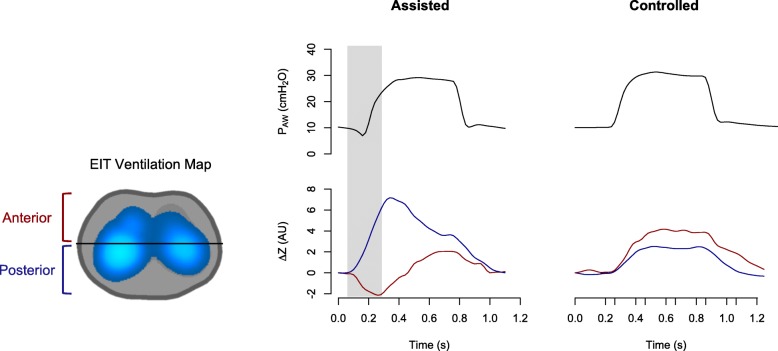
Pendelluft phenomenon. Variation of impedance (∆*Z*) and airway pressure in assisted and controlled mechanical ventilation (*P*_AW_). Blue line: posterior region of lung. Red line: Anterior region of lung. In assisted mechanical ventilation, anterior region of lung decreases its impedance variation (loses air) and at the same time posterior region increases (being aerated). AU arbitrary units, EIT electrical impedance tomography

Pendelluft is an intrapulmonary dyssynchrony described in the presence of intense diaphragmatic contraction, in which there is gas movement between different pulmonary regions (Fig. 10) [47]. Pendelluft causes tidal recruitment of dependent regions (local atelectrauma) by concomitant deflating nondependent regions. This transferred volume also causes excessive stretching of the alveoli in the dependent region (local volutrauma). Both injurious mechanisms worsen local pulmonary inflammation [48]. This regionally amplified transpulmonary pressure due to a strong inspiratory effort is usually undetected. The clinical hazard related to such amplification effect is “hidden” as the ventilators only measure airway-opening pressures. Only EIT is capable of detecting, tracking, locating, and quantifying pendelluft continuously and at the bedside.

### Pulmonary perfusion

**Fig. 11:**
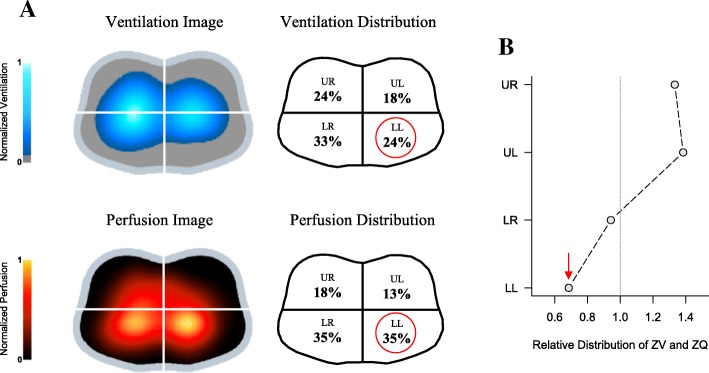
Electrical impedance tomography (EIT) ventilation and perfusion images of patient with community-acquired pneumonia affecting left lower lobe. Color scale adjusted by linear normalization. **a** Ventilation reduction at lower left quadrant in comparison with lower right quadrant, without changes in perfusion distribution at the lower quadrants. **b** Ventilation and perfusion decoupling in left lower quadrant represented by low distribution ratio. LL lower left, LR lower right, UL upper left, UR upper right, ZV ventilation estimated by EIT, ZQ perfusion estimated by EIT. Image provided by Fernando Suarez-Sipmann. Red arrow indicates ventilation/perfusion ratio in the LL quadrant

One of the targets of mechanical ventilation is to promote adequate gas exchange, but the efficiency of this process depends not only on ventilation but also on adequate pulmonary perfusion. Interestingly, EIT also estimates perfusion disturbances at the bedside. Lung perfusion assessment by EIT has been acquired using two methods: first-pass kinetics, performed by a brief respiratory pause, followed by a rapid intravenous bolus of hypertonic sodium chloride injected through a central venous line (the saline will act as an intravascular contrast due to its high conductivity) (Fig. 11) [49, 50]; and based on the separation of the cardiac signal to the ventilation signal by electrocardiography gating or by algorithms based on principal component analysis [51, 52].

Frerichs et al. [49] studied the efficacy of the EIT first-pass contrast method in an animal model of normal perfusion, simulating the presence of a pulmonary thromboembolism (by occlusion of the pulmonary artery through a Swan–Ganz catheter), and compared this with electron beam CT. The authors managed to generate new images for the distribution of the pulmonary regional blood flow with a good correlation with the data delivered by electron beam CT, concluding that EIT is able to detect alterations in the pulmonary perfusion and its changes in time. Later, the same group developed the pulmonary pulsatility method, which uses a frequency filter to separate the ventilation and perfusion components of the global impedance signal. This tool can be applied to assess functional changes in pulmonary perfusion such as the activation of hypoxic pulmonary vasoconstriction in one-lung ventilation [53].

### EIT-based indices/indices developed from EIT

The images of different patients obtained from EIT cannot be compared directly with each other, since the technique delivers only relative values (aeration changes from a variable reference image). To quantify and be able to compare the findings obtained from EIT, different indices have been developed from the “offline” analysis of the data. Next, we will present and explain three of the most used indices in the literature: center of ventilation (CoV), global inhomogeneity index (GI), and regional ventilation delay (RVD).

#### Center of ventilation

In 1998, Frerichs et al. [54] developed the concept of “geometric center of ventilation”. This parameter describes the variations of the pulmonary ventilation distribution in the ventral–dorsal direction and was defined mathematically as a vertical coordinate that marks the point where the sum of the regional ventilation (ventral and dorsal) divides the lung into two equal parts. Subsequently, the same group studied by EIT an experimental model of neonatal acute pulmonary injury due to depletion of surfactant, observing that the induction of acute lung injury displaced the ventilation center from the dependent to the nondependent regions. Accordingly, the application of recruitment maneuvers and surfactant administration moved the ventilation center back to the dependent regions, homogenizing the distribution of ventilation [55].

Recently, Sobota and Roubik [56] proposed a modification in the method to calculate the ventilation center from EIT, using an image segmentation method, according to the following equation:

$$ \mathrm{CoV}=\frac{n+K+0.5}{N+1}, $$

where *N* represents the total number of pixels of the tidal image, *n* indicates the number of the row of pixels where the sum of each of them is less than 50 ($\sum \limits_{i=1}^n$*r*_*i*_ ≤ 50), and *K* corresponds to a correction in the estimation of the ventilation center, in case it is between two pixels [56]:

$$ K=\frac{50-\sum \limits_{i=1}^n\ {r}_i}{r_n}. $$

#### Global inhomogeneity index

Zhao et al. [57] studied the global and regional heterogeneity of the volume distribution within the pulmonary parenchyma. For this, they developed an index that measures the impedance variations of each pixel between the end of inspiration and expiration (tidal or functional image). In practice, the GI index is calculated as the sum of the impedance changes of each pixel with respect to its median (in absolute values), divided by the sum of the impedance values of each pixel, which allows the index to be applicable to comparisons between individuals:

$$ \mathrm{GI}=\sum \limits_{x,y\in \mathrm{lung}}\ \left|D{I}_{xy}-\mathrm{median}\ \left(D{I}_{\mathrm{lung}}\right)\right|/\sum \limits_{x,y\in \mathrm{lung}}\ D{I}_{xy}. $$

DI indicates the value of the differential impedance in the tidal images; DIxy is the pixel in the identified lung area; DI_lung_ are all the pixels in the lung area. Subsequently, the same group demonstrated in 50 subjects connected to mechanical ventilation that the GI index allows one to indirectly quantify the heterogeneity of ventilation and also allows comparing these results between individuals [58].

In another context, Bickenbach et al. [59] evaluated the utility of the GI index to predict failure of a spontaneous breathing trial (SBT). They evaluated 31 tracheostomized patients with difficult weaning connected to mechanical ventilation in the pressure support mode. When comparing measurements at baseline, during (30 min), and after (120 min) a spontaneous breathing trial with a T-tube, they found a progressive increase in pulmonary inhomogeneity over time. The authors reported that patients who started the SBT with high GI values presented a higher probability of failing the SBT, concluding that analysis of the GI index could be a useful tool to predict failure of SBT in high-risk patients. Zhao et al. [58] performed a retrospective study where they used the GI index to choose the best PEEP value, defined as the PEEP level where lungs were most homogeneously ventilated. The chosen value was compared with the pulmonary compliance methods and PV curves. They concluded that this optimal PEEP can be identified using the GI index.

#### Regional ventilation delay index

**Fig. 12:**
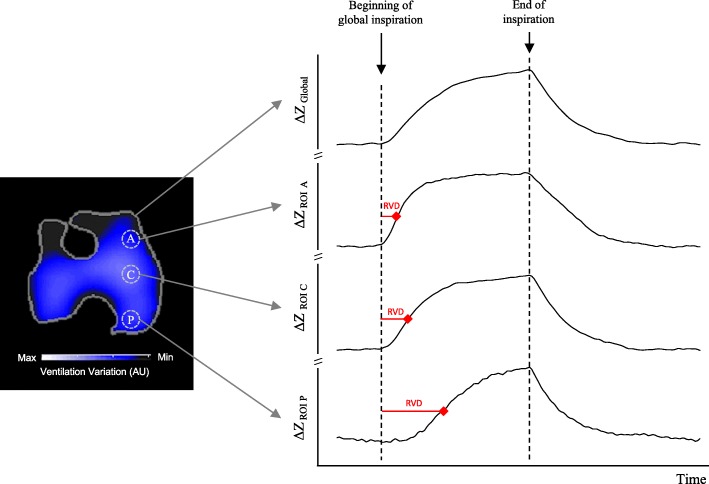
Regional ventilation delay (RVD). Ventral region. Patient in mechanical ventilation. Slice 1, ventral region; Slice 2, central ventral; Slice 3, central dorsal; Slice 4, dorsal region. A anterior, AU arbitrary units, C central, P posterior, ROI region of interest, ∆*Z* variation of impedance. Courtesy of Wildberg Alencar

Wrigge et al. [60] evaluated the utility of EIT to estimate regional ventilation and alveolar recruitment. For this, they simultaneously compared EIT acquisitions with dynamic CT images. To determine the potential for alveolar recruitment, they evaluated the delay time of ventilation in certain ROIs in two experimental models of acute lung injury (acid aspiration plus abdominal hypertension and injection of oleic acid). By means of a mathematical analysis of the regional impedance–time curves, the delay time was estimated between the beginning of the inspiration until the slope of the impedance/time curve reached a certain percentage of the inspiratory time in comparison with the global image (Fig. 12). They demonstrated that the RVD index correlated well with the estimated alveolar recruitment seen in the CT images (*R*^2^ > 0.6):

$$ {\mathrm{RVD}}_i=\left(\Delta  {t}_{\mathrm{RVD}}/\Delta  {t}_{\max -\min}\right)\times 100. $$

Muders et al. [61] evaluated RVD to quantify the recruitment caused by different levels of PEEP in an experimental model of acute lung injury by injection of oleic acid and abdominal hypertension. The animals were connected to mechanical ventilation with different levels of PEEP (0, 5, 10, 15, 20, and 25 cmH_2_O) in a randomized manner. The RVD index was used to quantify the time it took for certain lung regions (quadrants and pixels) to reach a certain threshold of impedance change. From this index, the authors developed a regional ventilation delay inhomogeneity, which quantifies the temporal heterogeneity of ventilation (calculated from the deviation of RVD index values of each pixel). When comparing this index with the potential for recruitment estimated by CT images, a moderate linear interindividual relationship was observed.

Bickenbach et al. [59] also studied the usefulness of RVD during a SBT in patients with difficult weaning, calling this new variant the regional ventilation delay index during spontaneous breathing (spRVD), suggesting that this could be a significant tool for the evaluation of pulmonary heterogeneity in patients during a SBT.

## Conclusion

Lung EIT is a promising clinical tool for continuous and real-time monitoring of pulmonary ventilation that can be especially useful in severe mechanically ventilated patients such as those with ARDS. EIT can help to optimize mechanical ventilation settings, detect complications such as derecruitment and pneumothorax, and provide estimates of perfusion distribution. More clinical validation studies are awaited to explore the full potential of the technology.

## Abbreviations

∆*Z*: Delta *Z*
ARDS: Acute respiratory distress syndrome
CoV: Center of ventilation
CT: Computed tomography
EELV: End-expiratory lung volume
EELZ: End-expiratory lung impedance
EIT: Electrical impedance tomography
FRC: Functional residual capacity
GI: Global inhomogeneity index
ICU: Intensive care unit
PEEP: Positive end-expiratory pressure
*P*_plateau_: Plateau pressure
PV: Pressure–volume
ROI: Region of interest
RVD: Regional ventilation delay
SBT: Spontaneous breathing trial
SPECT: Single photon emission computed tomography
SpRVD: Regional ventilation delay during spontaneous breathing
VILI: Ventilator-induced lung injury
